# ISCEV extended protocol for the stimulus–response series for the dark-adapted full-field ERG b-wave

**DOI:** 10.1007/s10633-019-09687-6

**Published:** 2019-03-30

**Authors:** Mary A. Johnson, Brett G. Jeffrey, André M. V. Messias, Anthony G. Robson

**Affiliations:** Department of Ophthalmology and Visual Sciences, University of Maryland School of Medicine, 10 S. Pine St., MSTF Suite 500-A, Baltimore, MD 21201, USA; Ophthalmic Genetics and Visual Function Branch, National Eye Institute, Bethesda, MD, USA; Oftalmologia e Otorrinolaringologia e Cirurgia de Cabeça e Pescoço, School of Medicine of Ribeirão Preto, University of São Paulo, Ribeirão Preto, Brazil; Department of Electrophysiology, Moorfields Eye Hospital, London, UK; Institute of Ophthalmology, University College London, London, UK

**Keywords:** Clinical standards, Electroretinogram (ERG), International Society for Clinical Electrophysiology of Vision (ISCEV), Intensity–response, Retinopathy, Naka–Rushton

## Abstract

The International Society for Clinical Electrophysiology of Vision (ISCEV) standard for full-field electroretinography (ERG) describes a minimum protocol for clinical testing but encourages more extensive testing where appropriate. This ISCEV extended protocol describes an extension of the ISCEV full-field ERG standard, in which methods to record and evaluate the growth of the dark-adapted (DA) ERG b-wave with increasing stimulus energy are described. The flashes span a range that includes the weakest flash required to generate a reliable DA ERG b-wave and that required to generate a maximal b-wave amplitude. The DA ERG b-wave stimulus–response series (also known historically as the “intensity–response” or “luminance–response” series) can more comprehensively characterize generalized rod system function than the ISCEV standard ERG protocol and may be of diagnostic or prognostic value in disorders that cause generalized rod system dysfunction.

## Introduction

The International Society for Clinical Electrophysiology of Vision (ISCEV) standard for full-field electroretinography (ERG) describes a minimum set of tests but encourages the use of additional ERG protocols for clinical ERG testing [1]. This extended protocol describes the flash stimulus–response series for the dark-adapted (DA) ERG b-wave amplitude, referred to as the “intensity–response” or “luminance–response” function in many published studies. The protocol is a specialized procedure that is well established and broadly accepted by experts in the field and was prepared by the authors in accordance with ISCEV procedures (http://www.iscev.org/standards/index.html#guide2procedures). The protocol was approved by the ISCEV Board of Directors on February 27, 2019.

## Scope and applications

**Fig. 1:**
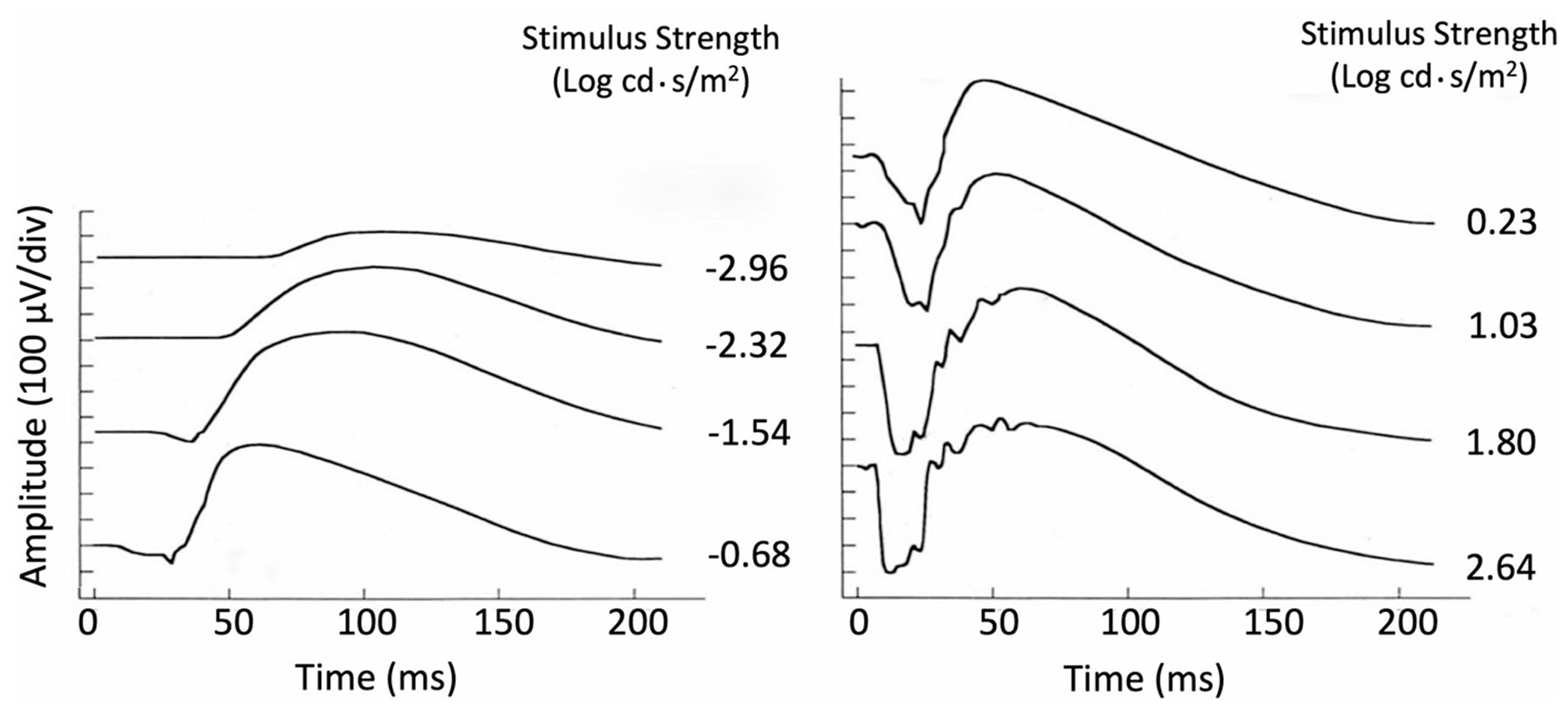
Human DA ERG waveforms recorded to flashes of increasing strength (intensity). After: Johnson (1991) in Heckenlively and Arden [2]

The ERG is a graded response whereby amplitude, timing and waveform change with increasing stimulus strength (Fig. 1). This protocol describes the process of recording DA ERGs using a series of increasing stimulus strengths and of analyzing the data by their fit to a heuristic model. The derived parameters of the model characterize the maximal rod-mediated retinal response and provide a measure of retinal sensitivity that may aid understanding of pathophysiology in some retinopathies.

Naka and Rushton derived the stimulus–response (V–log I) function, expanded by others and detailed below, by recording intra-retinal voltage (S-potentials) in fish to a range of light stimuli [3]. The stimulus–response characteristics of human DA ERG b-waves were first described by Fulton and Rushton [4], who fit b-wave amplitude data with a saturating hyperbolic (H2) function. Later, the DA ERG stimulus–response relationship was refined in a letter to the editor by Massof and Johnson [5] that specifically documented an exponentiated form of a hyperbolic relationship. This function was referred to as the so-called Naka–Rushton equation in a 1984 study of ERGs in retinitis pigmentosa [6], and this name was retained in most subsequent studies of the ERG b-wave stimulus–response function (but see “Technical Issues: f. Nomenclature”).

**Fig. 2:**
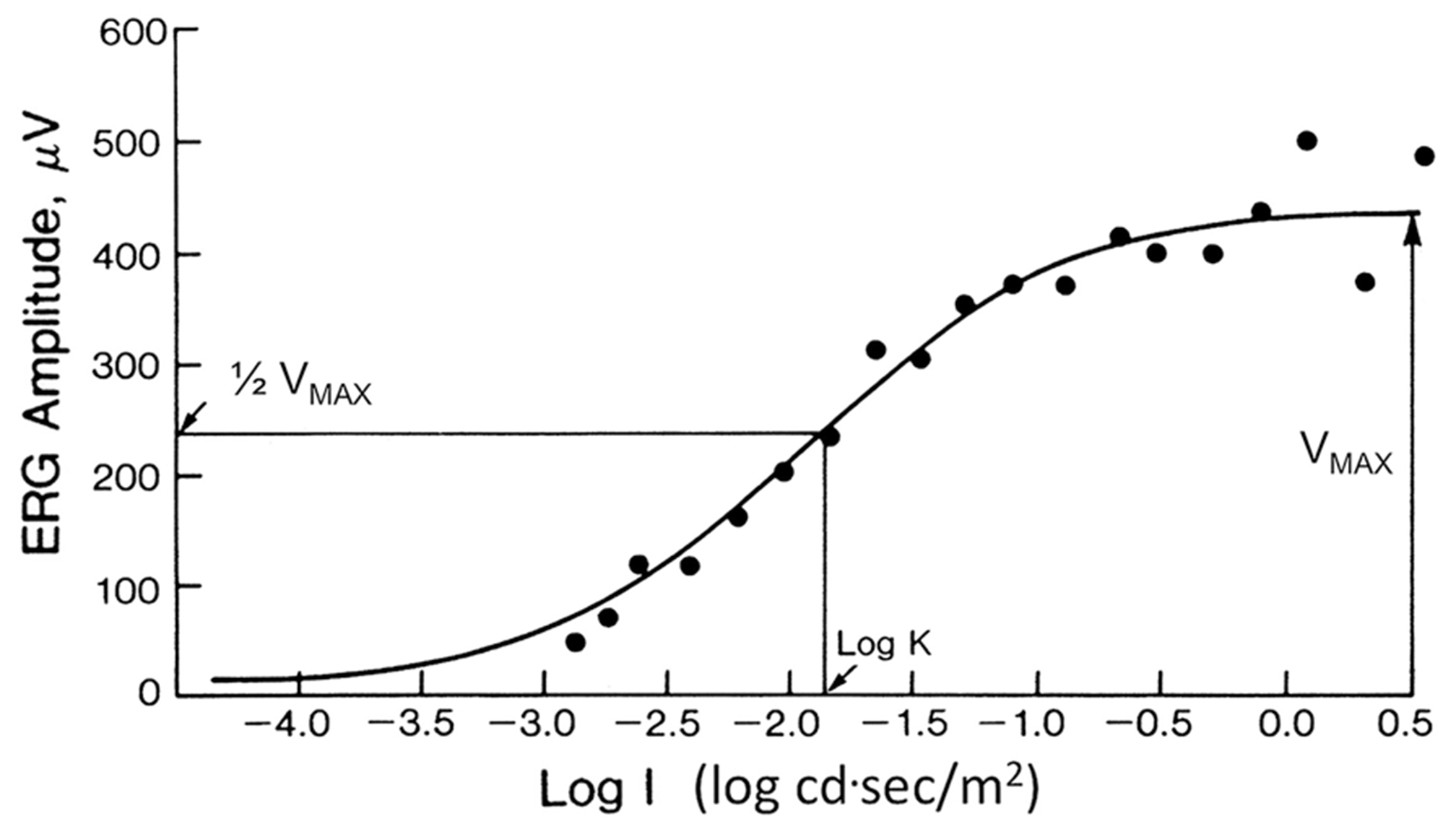
An example of flash stimulus–response amplitude data for DA ERG b-waves recorded from a healthy subject to flashes of increasing strength (intensity, I)

The flash stimulus–response series for the DA ERG b-wave amplitude may be described reasonably well by the following function:

$$V=V_{\max}I^{n}/I^{n}+K^{n},$$

where *V* (μV) is the ERG b-wave amplitude generated in response to flash intensity *I* (cd·s/m^2^). The derived parameter *V*_max_ (μV) is the asymptotic amplitude of the function, *K* (cd·s/m^2^) is the flash intensity that elicits a response that is ½ V_max_, and *n* is a dimensionless number representing the slope of the curve and generally considered to equal 1. An exception to the approximation of *n* as 1 occurs in disorders producing marked heterogeneity in retinal sensitivity, such as retinitis pigmentosa (RP). In these disorders, “*n*” can be much < 1, and thus, it is necessary to allow “*n*” to vary in order to achieve a good approximation to the data. *V*_max_ has been interpreted as an index of both the number of rods responding and the gain (μV/quanta) for each b-wave generator. A reduction in *V*_max_ can result from loss of photoreceptors, disruption of the dark current, inner retinal dysfunction or some other types of response compression. The parameter *K* has been interpreted as an index of retinal sensitivity that represents the efficiency of quantal capture. An increase in *K* would shift the entire function to the right, indicating that a stronger stimulus is required to elicit b-waves of comparable amplitude. Reductions in *V*_max_ and increases in *K* may be seen individually, or more frequently, in combination in many retinal diseases. Figure 2 is an example of b-wave amplitude data recorded as a function of log flash intensity in a normal human observer.

Stimulus–response function parameters *V*_max_ and *K* may be used to obtain additional information about the etiology or prognosis in a number of disorders. Such parameters have helped characterize fundamental differences between the mechanisms of rod dysfunction and degeneration in rod–cone dystrophy (RP) and cone–rod dystrophy [7]. Patients with RP typically show a loss in *V*_max_ along with an increase in *K*, whereas patients with cone–rod dystrophy usually show normal values for these parameters. Exceptions include KCNV2 retinopathy (“cone dystrophy with supernormal rod ERG”), characterized by generalized cone system dysfunction, pathognomonic DA ERG changes and an abnormal ERG stimulus–response series [8, 9].

**Fig. 3:**
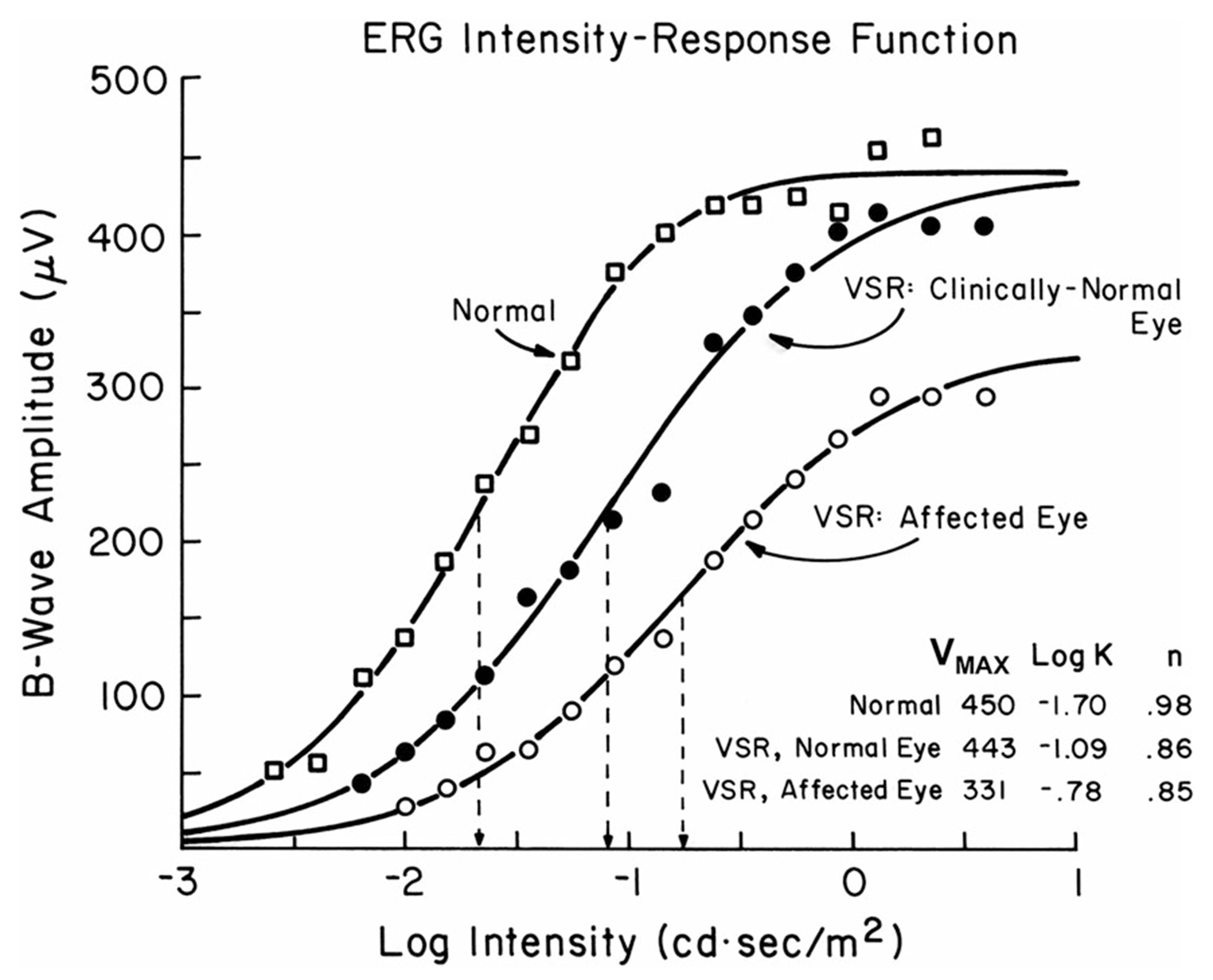
DA ERG b-wave stimulus–response functions in the affected and clinically normal eyes of a patient with venous stasis retinopathy (VSR), compared to an age-similar healthy subject

Figure 3 shows an illustrative example of a normal stimulus–response function compared with one from a case of venous stasis retinopathy (VSR). There is loss in *V*_max_ in the affected eye of 0.13 log, but an increase in *K* of 0.92 log. The fellow eye also showed a large increase in *K* (0.61 log) with a normal *V*_max_, highlighting the possibility of subclinical involvement.

Stimulus–response functions also have been used to evaluate the timeline of retinal development and aging [10–13], and toxicity and efficacy in pharmaceutical studies [14–18]. They have been recorded in many degenerative retinal disorders [6, 7, 19–27], as well as in congenital stationary night blindness (CSNB), in which they reveal differences between the complete and incomplete forms [28]. The function has also been useful in other disorders such as age-related macular degeneration (AMD; [29]), altitude retinopathy [30], central retinal artery and vein occlusions [31–36] and diabetic retinopathy [37–39].

Patients who have an increase in log *K* will also have a delay in b-wave timing because peak times change with stimulus strength. ERG peak time measurements can be used to estimate retinal sensitivity loss and have been used to predict proliferative retinopathy in CRVO [31, 35, 36] and diabetic retinopathy [37, 39].

## Patient population

Patients of all ages, able to tolerate ganzfeld stimulation, are referred for investigation of rod-mediated retinal function. Using this paradigm, patients with selective cone-mediated abnormalities will usually show minimal changes in the derived parameters produced by the curve fit to the data [7].

## Technical issues

This protocol has the same requirements as those outlined under the basic technology section of the ISCEV ERG protocol [1]. Additional considerations are outlined below.

**Fig. 4:**
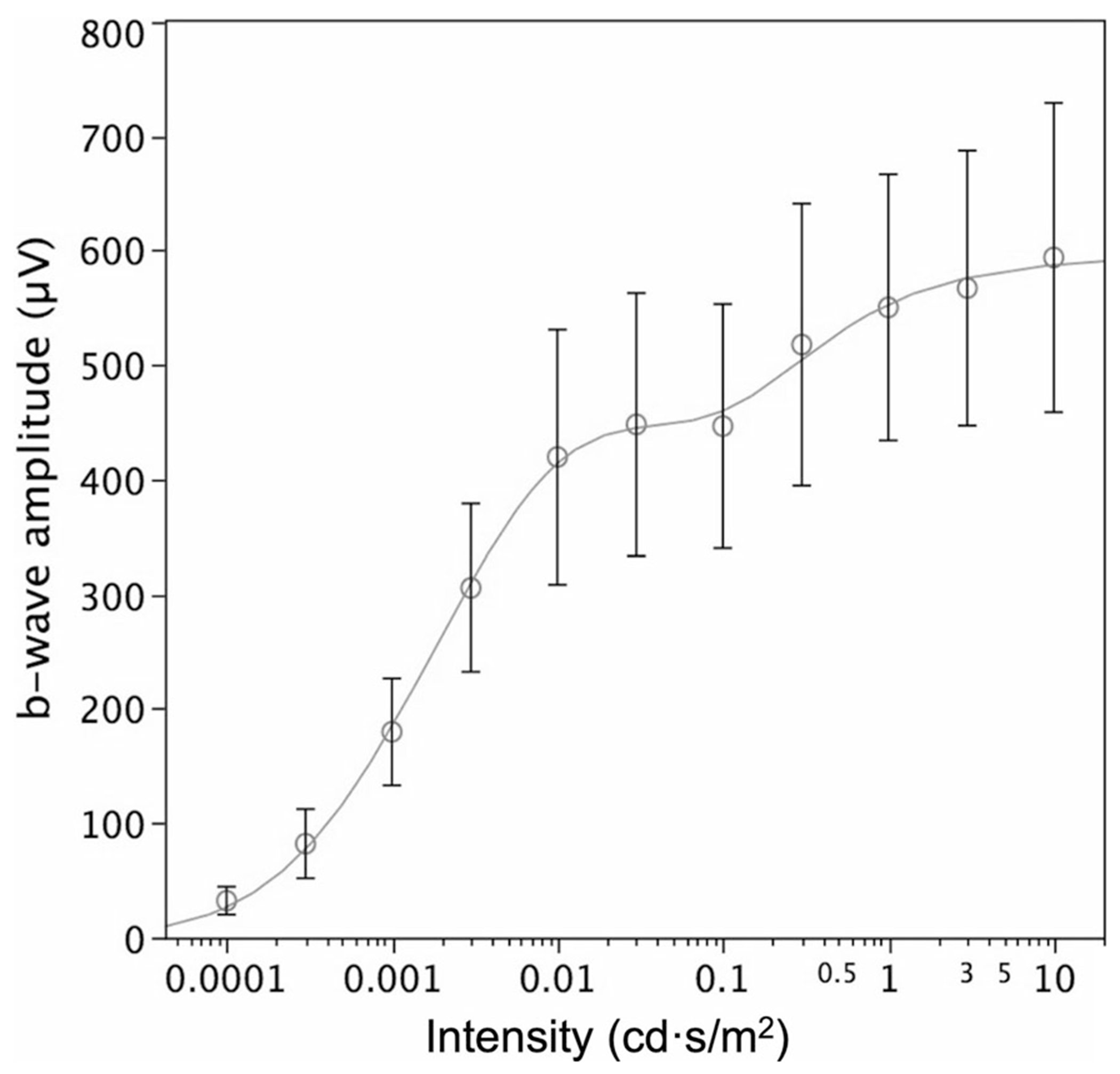
An example of flash stimulus–response amplitude data for DA ERG b-waves averaged from 85 healthy subjects to flashes of increasing strength (intensity). Circles are averages, error bars the 95% confidence limits, and the line a mathematical model that includes the second limb, seen in all normal subjects tested above flash strengths of approximately 0.1 cd·s/m^2^

- Range of flash strengths
  To adequately characterize the stimulus–response series, flash stimuli must span a range that includes the dimmest flash required to generate a reliable DA ERG b-wave and that required to generate a maximal b-wave amplitude. This normally occurs over a flash range of 3.5 to 4 log units.
- Inter-stimulus interval
  The inter-stimulus interval should be sufficiently long to maintain the same level of dark adaptation throughout the procedure. The ISCEV ERG standard specifies an inter-stimulus interval of 2 s for DA 0.01 and 10 s for DA 3.0, but there are no specific recommendations for stimuli between these two stimulus strengths. It is recognized that the stimulus–response series will asymptote at stimuli well lower than DA 3.0 for most individuals.
- Amplifier gain
  Amplifier gain will need to be higher for the dim stimuli and should be increased until responses can be seen well enough to judge reliability.
- Signal quality
  Waveforms may be small or of long peak time and prone to noise or intrusion of blink and eye movement artifacts [40].
- Fitting the series
  Near the asymptote of the stimulus–response function, a second hyperbolic function can be seen in normal subjects (Fig. 4). By showing that this is observed in rod monochromats, Peachey et al. [41] suggested that the second limb did not result from an interaction between rod and cone systems but is more likely to represent interference between the processes responsible for the a- and b-waves, since b-wave amplitudes are measured from the trough of the a-wave to the peak of the b-wave.

**Fig. 5:**
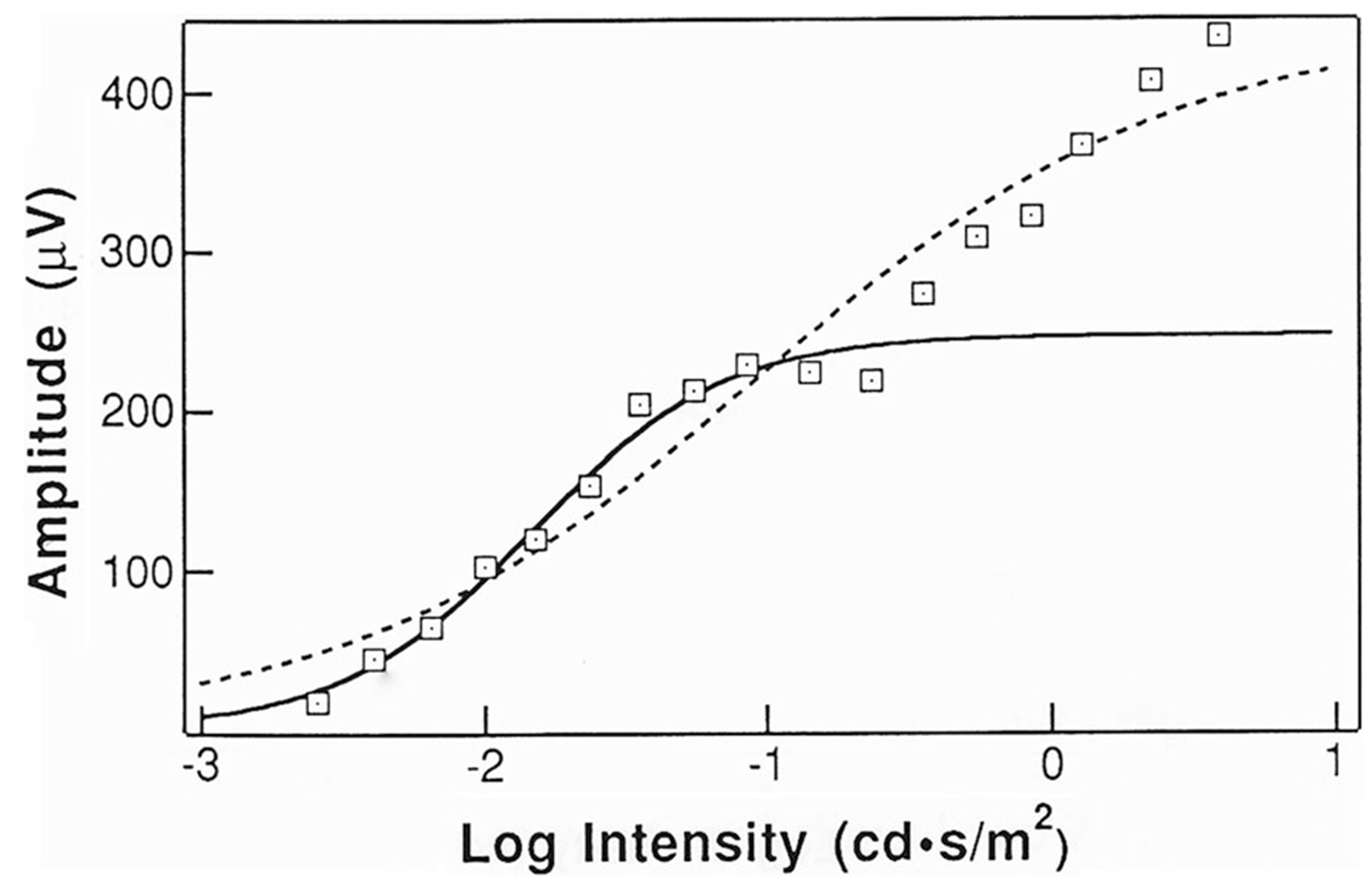
Example of ERG b-wave stimulus–response data, fit by single Naka–Rushton equations to all of the data (dashed line) and to just the first limb (solid line). Figure reprinted with permission of Springer Nature (Documenta Ophthalmologica 85 [2] pp. 135–150). The care and fitting of Naka–Rushton functions to electroretinographic intensity–response data (Severns ML, Johnson, MA, Copyright 1993) [42]

The occurrence of a second limb may confound routine application of a single hyperbolic stimulus–response function and would result in a spuriously high *V*_max_ and an elevated log *K*. The latter is illustrated in Fig. 5, which shows a single hyperbolic function fit to data obtained from one subject. When all of the data are included (dashed line), estimates of *V*_max_ increased from 233 to 438 μV, and the corresponding log *K* estimates increased from − 1.89 to − 1.03, when compared to the parameters obtained from fitting just the first limb (solid line). Thus, in most patients, use of strong flashes will confound analysis unless the second limb is identified.

A heuristic method for identifying and excluding the second limb has been described [42]. The optimal stimulus increment size for recognizing the second limb was 0.4 log unit up to about the point the b-wave begins to grow rapidly, and 0.2 log unit steps afterward. The smaller increment is necessary to recognize the occurrence of a second limb when it exists. The second limb can also be excluded manually, i.e., those data points that do not form a part of the single hyperbolic function can be omitted prior to curve fitting.

(f) Nomenclature
  In seminal studies and in most relevant publications, the light stimulus–response function is referred to as the ERG “intensity–response” function. The term “luminance–response” function has also been used. It is acknowledged that flashes should be described in terms of energy (luminous energy per unit solid angle per unit area) rather than intensity, but this and the widely used term “intensity–response” are retained in reference to historical data and to the parameter (I) used in the equation. The term “Naka–Rushton” equation is commonly used to describe the DA ERG b-wave amplitude stimulus–response function mathematically, but the eponym is difficult to justify based on the original studies that described a more basic equation (see above).

## Calibration

All stimuli should be individually calibrated and rechecked over time following the current ISCEV standard and guidelines [1]. Nominal flash strengths or nominal increments in flash stimuli should not be used. Standard ERG stimuli may have a very short duration (10 μs for xenon bulbs), so a calibration device that can time-integrate flashes is required. Dim flashes from a xenon source generally vary more than flashes from a light-emitting diode (LED) and may require assessment of multiple flashes to measure mean flash intensity. LED sources can produce more reliable flash stimuli because their output is determined by the current applied to them. Most conventional ERG equipment that is manufactured currently uses LEDs, which produce more stable flash intensities.

## Protocol specifications

This protocol follows the same procedures for patient preparation and recording that are outlined under the clinical protocol section of the ISCEV standard full-field ERG protocol [1]. Other specifications are listed below.

- Flash strength
  Flash stimuli should span a range of approximately 3.5 to 4.0 log units. In the absence of retinal dysfunction, the typical range would be approximately − 3.5 to 0.5 log cd·s/m^2^, starting with the lowest flash strength that will generate a reliable DA ERG b-wave up to that required to elicit a maximal b-wave. Initially, stimuli should be recorded in increasing steps of about 0.4 log units, until the b-wave amplitude begins to grow rapidly (near log *K*). Thereafter, step sizes should be reduced to 0.2–0.3 log units to enable detection of the second limb. Thus, at a minimum, about a dozen points are required to characterize the stimulus–response series in the absence of dysfunction.
- Inter-stimulus interval.
  To avoid light adaptation, this protocol specifies a time between flashes of 2 s for stimuli up to 0.01 cd·s/m^2^, 3 s for stimuli up to 0.1 cd·s/m^2^ and 5 s for stimuli up to 3 cd·s/m^2^. For most patients, the stimulus–response function will asymptote at values between 0.1 and 1.0 cd·s/m^2^.
- Amplifier gain.
  There is no specific requirement for amplifier gain except that it needs to be high enough to evaluate the waveform and may need to be increased for responses to dim stimuli.
- Signal averaging.
  Individual ERG waveforms should be assessed for repeatability, and inconsistent or artifactual waveforms should be eliminated before averaging. If signal averaging is needed, 3 to 10 sweeps are usually sufficient.

## Response evaluation

The DA ERG b-wave amplitudes should be measured as described in the ISCEV ERG standard [1]. The b-wave amplitudes at different flash strengths should then be input into one of the many commercially or privately available computer programs that provide a fit to the data using a suitable mathematical stimulus–response function (as above). Many of these programs use the “Michaelis–Menten” equation for enzyme kinetics. (The “Michaelis–Menten” equation is the same as the “Naka–Rushton” equation except that the former sets “*n*” to 1.) The program used, in addition to fitting a curve to the data, should provide estimates of the maximum amplitude (*V*_max_) and the semi-saturation constant (*K*). The plot of b-wave amplitude versus flash strength should be visually examined for goodness of fit. If a second limb is present, the computer fit to the data should be adjusted either by omitting the points on the second limb and refitting the data, or by using a heuristic method that fits both limbs and eliminates the second [42]. As mentioned above, significant retinal heterogeneity will reduce “*n*,” and an equation that allows all the three parameters to vary may be necessary to obtain a good fit to the data.

## Reporting

Use of the ISCEV extended stimulus–response protocol for the DA ERG b-wave should be acknowledged, and any departures from the ISCEV standard ERG methods or extended protocol should be stated. Any technical or compliance difficulties such as excessive eye movements or eye closure should be noted.

Parameters *V*_max_ and log *K* should be reported, as well as normal ranges for fully dilated eyes. It is recognized that for some applications, a qualitative description of the stimulus–response series may be sufficient to corroborate or suggest a diagnosis. Eyes with smaller pupils will have an increasingly dimmer retinal illuminance (measured in Trolands), which will affect the value of log *K*. For this reason, pupil size should always be measured. Compensation for light attenuation from small pupils is possible using Table 1, and any correction to log *K* should be stated.

## Figures and Tables

**Fig. 6:**
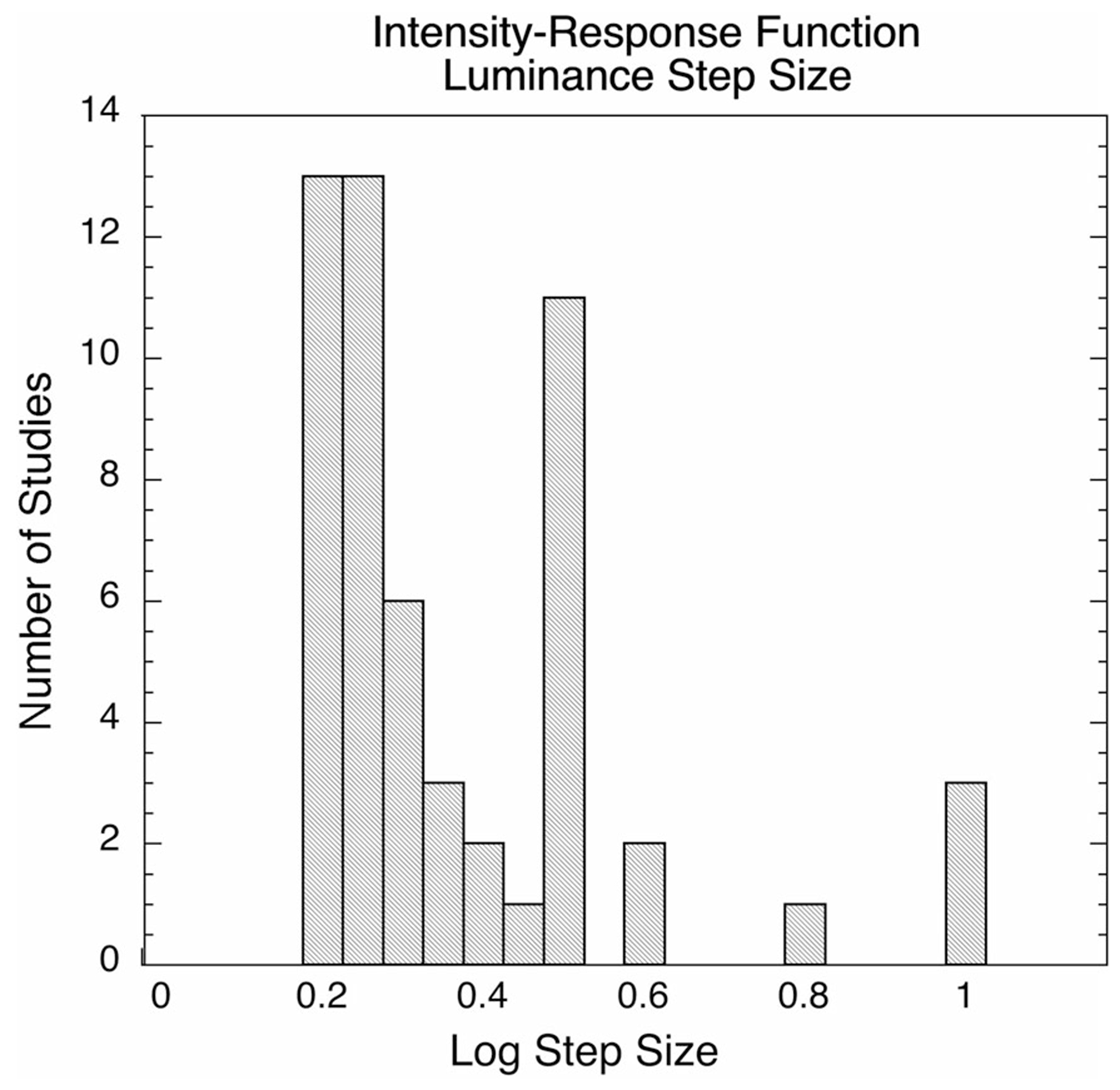
Flash strength step sizes (increments) used for collection of ERG “intensity–response” data

**Table 1:** Effect of pupil size on estimates of log *K*

Pupil diameter (mm)	Pupil log area	Number to be added to the value of log *K*
8	1.70	0
7	1.59	− 0.11
6	1.45	− 0.25
5	1.29	− 0.41
4	1.10	− 0.60

**Table 2:** Literature used for analysis

Citations	Flash strength range	Luminance step size	Method of fitting	Patient population
[32]	6 log (− 2.58 to 3.58 log cd·s/m^2^)	0.5 log	None	Central retinal artery occlusion
[6]	3 log	15 steps (0.2 log)	Naka–Rushton	RP
[43]	5 log	11 steps (0.5 log)	Logistic function	27 patients after renal transplant
[29]	3.7 log (− 2.9 to 0.6 log cd·s/m^2^)	15 steps (0.25 log)	Naka–Rushton	AMD
[44]	3.25 log		Naka–Rushton	
[19]	4.0 log (− 2 to 2 log scot td-s)	16 steps (0.25 log)	Naka–Rushton	RP
[45]	3.5 log	6 steps (0.6 log)	Naka–Rushton	Isotretinoin toxicity
[20]	7.5 log	0.5 log or 1.0 log	none	Supernormal rod ERG
[21]	4.1 log (using rod isolation)	0.2 log per step	Naka–Rushton	RP
[7]	4.1 log (using rod isolation)	0.2 log per step	Naka–Rushton	RP, cone dystrophy
[22]	4.1 log (using rod isolation)	0.2 log per step	Naka–Rushton	RP
[23]	3.5 log (− 2.61 to 0.87 log cd·s/m^2^)	18 steps (0.2 log)	Naka–Rushton	X-linked RP
[33]	4 log	8 steps (0.5 log)	Naka–Rushton	CRVO
[41]	4 log	17 steps (0.25 log)	Naka–Rushton	Normal and achromatopsia
[46]	3.8 log (− 2.97 to 0.82 log cd·s/m^2^)	18 steps (0.2 log)	Naka–Rushton	Albinism
[47]	4 log	13 steps (0.3 log)	Naka–Rushton	Cone–rod degeneration
[48]	3.8 log (− 2.97 to 0.82 log cd·s/m^2^)	18 steps (0.2 log)	Naka–Rushton	Sickle cell retinopathy
[24]	3.5 log (− 1.5 to 2 log scot td-s)	13 steps (0.25 log)	Naka–Rushton	Elevated cyclic Guanosine monophosphate type Human retinal degeneration
[34]	3.6 log	8 steps (0.6 log)	Naka–Rushton	Central retinal vein occlusion
[49]	4.5 log (− 3.6 to 0.9 log cd·s/m^2^)	10 steps (0.45 log)	Naka–Rushton	Normal
[50]	5 log (− 1 to 4.0 log scot td-s)	19 steps (0.26 log)	Naka–Rushton	
[51]	3.6 log	13 steps (0.27 log)	Naka–Rushton of first limb	Normal
[35]	4 log	0.2 log per step	Naka–Rushton	Central retinal vein occlusion
[36]	Threshold to 0.29 log cd·s/m^2^	0.2 log	Naka–Rushton	Central retinal vein occlusion
[10]	2.5 log (− 2.0 to 0.5 log scot td-s)	0.28 log	Naka–Rushton	Normal over the life span
[25]	3.25 log (− 1.19 to 2.04 log scot td-s)	Unspecified	Naka–Rushton	RP and normal
[14]	3.50 log (− 4.5 to − 1 log cd·s/m^2^)	0.2 log per step	Naka–Rushton	Sildenafil toxicity
[52]	3.25 log (− 4.25 to − 1 log cd·s/m^2^)	14 steps (0.25 log)	Naka–Rushton	Normal
[53]	5.0 log (to 25.2 cd·s/m^2^)	0.3 (Boston site); 1 log (Cambridge site)	Naka–Rushton	Normal children and adults
[54]	3.7 log (− 0.7 to 3.0 log scot td-s) using rod isolation	Unspecified	Naka–Rushton	Cone dystrophy
[15]	3.50 log (− 4.5 to − 1 log cd·s/m^2^)	0.2 log per step	Naka–Rushton	Sildenafil toxicity
[55]	4.0 log (− 5.01 to − .96 log cd·s/m^2^) blue light	11 steps (0.36 log)	Naka–Rushton	Normal circadian rhythm
[56]	4.0 log (− 3.95 to 0.05 log cd·s/m^2^)	0.3 log;# of steps depending on the start of second limb	Naka–Rushton	Depression
[57]	3.25 log (− 4.25 to − 1.0 log cd·s/m^2^) green light	14 steps (0.23 log)	Naka–Rushton	Normal, dilated versus undilated
[58]	3.25 log (− 4.25 to − 1.0 log cd·s/m^2^) green light	14 steps (0.23 log)	None	Normal
[8]	4.06 log (− 3.0 to 1.06 log cd·s/m^2^)	8 steps (0.5 log)	None	KCNV2 retinopathy
[59]	3.25 log (− 4.25 to − 1.0 log cd·s/m^2^)	14 steps (0.23 log)	Sigmoid curve	Normal, patients with seasonal affective disorder
[11]	4.9 log (− 3.27 to 2.16 log scot td-s) using rod isolation	0.4 log	Naka–Rushton	Preterm infants with and without ROP
[16]	3.5 log (− 3.27 to 0.26 log cd·s/m^2^)	8 steps (0.5 log)	Naka–Rushton	Toxicity (bevacizumab)
[17]	4 log (− 3.62 to 0.38 log cd·s/m^2^)	4 steps (1.0 log)	Naka–Rushton	Patients (unknown diagnosis) receiving bevacizumab
[18]	4.9 log (− 1.95 to 2.95 log scot td-s)	0.3 log	Naka–Rushton	Normal
[9]	5 log (− 4 to 1 log cd·s/m^2^)	10 steps (0.5 log)	Naka–Rushton	KCNV2 retinopathy
[60]	5 log (− 4 to 1 log cd·s/m^2^)	10 steps (0.5 log)	Naka–Rushton	Normal
[4]	4.2 log (− 0.7 to 3.5 log scot td-s)	7 steps (0.5 log)	H2 clearance curves	Normal
[61]	5.5 log (− 4.5 to 1.0 log cd·s/m^2^)	0.5/0.25 log	Naka–Rushton	Glaucoma/normal (35/17)
[26]	3.5 log (− 1.5 to 2 log scot td-s)	13 steps (0.27 log)	Naka–Rushton	RP heterozygotes/normal (11/19)
[12]	4 log (− 3 to 1 log cd·s/m^2^)	0.25 (adult); 0.5 (infant)	Naka–Rushton	Infant ROP/adult normal (19/3)
[62]	3.9 log (− 3.95 to − 0.05 log cd·s/m^2^)	0.3 log	Naka–Rushton	Normal (10)
[27]	Not specified	Not specified	Naka–Rushton	RP/CRD/normal (11/17/50)
[38]	3.1 log (− 3.1 to 0 log cd·s/m^2^)	Variable (0.35 to 0.82 log)	Naka–Rushton	Diabetics/normal (65/10)
[42]	Threshold to 0.6 log cd·s/m^2^	0.2 log	Naka–Rushton	CRVO/normal (94/124)
[63]	3.8 log (− 3.0 to 0.8 log cd·s/m^2^)	18 steps (0.2 log)	Naka–Rushton	Normal (30)
[64]	3.0 log (− 2.9 to 0.1 log cd·s/m^2^)	0.3–0.4 log	Naka–Rushton	Normal (45: 61 eyes)
[13]	Not specified	7 steps	Naka–Rushton	Normal (269)
[39]	4.0 log (− 3.8 to 0.2 log cd·s/m^2^)	0.2 log	1. Naka–Rushton 2. NR to first limb 3. Log model (− 3.8 to − 2.2 log cd·s/m^2^)	Diabetics/normal (152/40)
[28]	5 log	0.3 log	Naka–Rushton	CSNB

## Appendix: Justification for the protocol details

A literature review was performed using the Medline search engine, for human studies that used the words ERG and intensity–response or Naka–Rushton or Michaelis–Menten or luminance–response. Studies dealing with the photopic hill, photopic negative responses (PhNRs), wavelets and multifocal ERGs were excluded. Table 2 shows the results of this search.

Of the 57 studies evaluated (Table 2), 60% used a flash intensity range of 3.5 to 4.5 log units (range 2.5–7.5). Forty-seven percent used a flash step size of either 0.2 or 0.25 log, but there was a spread of data, as shown in Fig. 6. The larger step sizes could easily misidentify a second limb unless weaker flashes were used. Most studies used the “Naka–Rushton” function to analyze the data.
